# The Hygiene of Motherhood

**Published:** 1891-03

**Authors:** John Sheppman


### THE HYGIENE OF MOTHERHOOD,

By Dr. John Sheppman.

### Part Fifth.

How to counteract the seeds of infection and to prevent the spread of contagious diseases, may be a serious problem among many young women ; and one, which, devoid of its technicalities, should be looked upon as an interesting study by every intelligent mother. There are few children who are fortunate enough to escape a siege of one of the many ills, and fewer parents who thoroughly realize the extent to which all sickness is more or less preventable. Science has now conclusively proven that all infectious, communicable, and contagious diseases are caused by a germ, or microbe, which multiplies with incredible rapidity as soon as it is taken into the human system. This minute living organism may be likened to a seed, or plant, which produces only after its kind, and which fructifies according to the cultivated, or favorable condition of the soil. That we are all in constant danger of pestilential infection, cannot be questioned after having microscopically examined all articles of food, drink, and the general condition of the atmosphere. Every thing seems to owe its production to some viable substance, which in its germinating process gives food, and takes food from that upon which it subsists. From these few lines, we can deduct the logical principles of the germ doctrine, which certifies that no inert matter has the power to proportionately increase itself, without some vital assistance.

If we allow our bodies to become corrupt, or undermined in health, we make of them an excellent breeding place for the seeds of all contagious diseases, which will take root, and propagate, in the most virulent manner. It is therefore very evident that the principal rules which we all should endeavor to follow, are, to keep the body in a clean and robust state, and to avoid doing any thing that has a detrimental influence upon our health. The small specks of living animal matter which float about in drinking water, are not all necessarily injurious, as a certain species must be present in order to bring about the dreaded results. No form of animal life can originate spontaneously. Nor can typhoid fever manifest itself without the presence of its specific germ. If we study the sources of infection, we will invariably find traces of some pre-existing case where antiseptic precautions were not rigidly followed. Past experiences have clearly demonstrated that all malignant epidemics have found their origin where the surroundings were in the most insalubrious condition. The prompt action of the health authorities, in such localities, does not always insure immediate benefit, as the devastating influences of a ravaging plague are too deeply seated to yield to the preliminary advances of sanitary laws. If every one would study the proper means of protecting *themselves*, there would be comparatively little dread of future epidemics, and all infectious diseases would unquestionably become historical facts of the past.

In speaking of prevention, it may perhaps be well to make a note of some of the prevalent diseases which are commonly called contagious. In this list must be mentioned: Small-pox (varioloid), diphtheria, scarlet fever (scarlet rash), typhoid fever, anthrax (glanders), erysipelas, yellow fever, consumption, cancer, measles, and leprosy. It must not be forgotten that each disease is caused by its own specific germ, and on that account, various methods are necessarily required in order to counteract them. The most important step to be taken to insure immunity from small-pox is, vaccination. Since its discovery by Jenner, the death rate from this disease has been remarkably lessened. Indeed, as late as 1850, nearly sixty per cent. of the people of England, were, to some extent disfigured by deep and lasting pockmarks. At the present time, not more than one per cent. are noticeable. It is now compulsory in England for every child over three months old to be vaccinated. This law is undoubtedly good; but an additional command should be enforced to compel people to re-vaccinate every five or seven years, as long as any evidence of the disease is present. If, after a second trial, the vaccination does not "take," it may be safely assumed that the person is properly protected, at least, for the time being. The cicatrix, or scar, upon those who have been vaccinated, should present a number of minute cavities, or a pitted appearance somewhat similar to the larger depressions which are always noticeable upon the face of an afflicted one. If this condition does not appear, you have good reasons for questioning the quality or the pureness of the lymph, with which the operation had been performed.

Small-pox patients should be particularly dreaded, as the crusts which fall from their skins, are thick with germs, that rise with the dust in the air, and instantly prove infectious. Every sufferer should be isolated as far as possible, and no one but the physician and nurse should be allowed to see them. After recovery, but before leaving the sick chamber, a daily bath should be taken in a tub containing about two gallons of hot water, mixed with a teacupful, or six ounces of the strong solution of carbolic acid. The scalp and hair should be thoroughly cleaned by immersing both, and by the vigorous use of a sponge or brush. If the patient dies, only those who are strong and well protected should go near the corpse. The body should be wrapped in cotton that has been previously saturated with a strong solution of carbolic acid. The funeral must always be private, and as soon after death as possible.

It may be well to mention that in all cases of contagious diseases, an apartment should be selected that can be thoroughly ventilated, and every article of furniture, bric-a-brac, pictures, and needless ornaments should be immediately removed. Lace curtains, in a room of infection, are extremely dangerous, as the germs will live, feast, and rally in their meshes. The nurse should be provided with a loose outer garment which she can put on, and take off, when entering, and leaving the room.

Microbes may be said to be peculiarly fastidious ; as each species is known to infest, and to thrive upon a certain organ of the body, and upon *that*, only. Thus the germs of small-pox appear upon the skin ; of scarlet fever and measles, upon the skin and throat. Of typhoid, yellow fever, and cholera, in the digestive canal. Each disease has its own imperial symptoms, which require treatment accordingly. The first duty of the physician and the housewife should be to investigate the cellar, and to have it put into a dry state of supreme cleanliness. The slop barrel in the yard should be placed in the farthest corner, and always tightly covered.

If diphtheria is in your midst, no person under twenty years of age should be allowed to go near the patient, nor to sleep in an adjoining room. The sufferer should be closely watched, and all matter that comes from the mouth and nose, should be caught in a pasteboard receptacle, and instantly destroyed by fire. Nothing should be taken from the room without being disinfected ; for this purpose, boiling water is recommended ; but very often a light solution of carbolic acid is preferable. All bowel evacuations should be submitted to a strong solution of sulphate of iron (two pounds to a gallon of water), thoroughly mixed, and allowed to stand for at least thirty minutes, before emptying. If the attendant is susceptible to the disease, an excellent precaution is to place a pinch of finely powdered boracic acid upon the tongue every two hours, and to swallow the spittle. This can be followed without the slightest fear of proving injurious.

The stools of all typhoid fever patients are the principal infectious substances, which must be thoroughly disinfected before leaving the room. A half-pint of the following solution, well mixed with the stool, and allowed to stand for about three-quarters of an hour, has always proved efficient. One gallon of warm water, with a teaspoonful of corrosive sublimate, stir well, and add two tablespoonfuls of common salt. The neglect of this measure has caused many deaths, as the germs from privy vaults have found their way into wells, poisoned drinking water, and have risen innumerable with the dried dust in the air. During my sojourn in this city (New Orleans), I have noticed that most of the people here use rain water, for drinking purposes. Contaminated water is the chief cause of typhoid, and this fact, no doubt, explains the remarkable absence in this vicinity of that disease.

Scarlet fever and scarlet rash are identically the same. One being a milder form of the other. Children who are afflicted with the "rash," should not be permitted to mingle with others, as many cases of severe fever, and even death have resulted therefrom. The after effects of this disease are noticeable in many asylums, where children who are deaf and dum, blind or imbecile are taken. Excepting vaccination, all the methods employed in cases of small-pox, are applicable in this fever. The exfoliation of the skin is particularly dangerous ; and the accumulations upon the sheets should be carefully gathered, and burnt. The convalescent should not be permitted to leave the room, until the peeling off of the skin has been completed. The disinfecting baths can be made a little weaker than those used for small-pox ; one ounce of carbolic acid, to a gallon of warm water, would be sufficient. If death occurs, a light solution of corrosive sublimate (twenty grains to a quart of warm water) should be used to sponge the body, after which it should be wrapped in cotton, sprinkled with carbolic acid. The funeral should always be private, and very soon after death.

All consumptives should occupy a room alone, thoroughly ventilated, and should understand the importance of properly disinfecting all sputa immediately after it has proceeded from the mouth. An excellent plan would be to expectorate in a paper bag, and to consign both bag and contents to the flames.

There are many incidents of fatal results attending attacks of measles, which should command a greater amount of care in all such cases. Isolation is compulsory; and disinfection of all mucus matter must not be neglected. Baths, like those recommended for scarlet fever convalescents, should be taken; but not until the cough and fever have entirely disappeared.

The contagiousness of cancer, need not be questioned; for many convincing proofs and experiments, are now established. The only precautions necessary are to avoid coming in contact with the affected parts.

In all cases of infection, every article taken from the sick room should first be plunged into a kettle of boiling water, and allowed to remain for a few minutes. This, of course, includes dishes and articles of every description. A strong solution of carbolic acid (one ounce to a quart of water) should always be kept in the room, to sponge bed linens; or any surface upon which mucus has accidentally fallen. After the disease has run its course, a system of disinfecting should at once be instituted. For this purpose it is best to use about three pounds of sulphur, in an iron pan, placed in the room upon bricks, over which should be poured a tablespoonful of alcohol. As soon as the room is tightly closed, the preparation should be lit, and allowed to burn, and fumigate, for at least five hours. Equal parts of carbolic acid and water, should be sprin kled all over the mattresses, and they should be hung out in the air for a day or two. The room must be allowed to remain open, day and night, for a week or ten days before re-occupying it. The floor must be scrubbed with hot water, containing a teaspoonful of corrosive sublimate, to every gallon of water, and a tablespoonful of salt. Carpets should be soaked in hot water, or sprinkled with the carbolic solution, and then hung out in the air. In cases of small-pox, it would be safer to fumigate with corrosive sublimate, following it, in three or four days, with sulphur.

In conclusion, I feel obliged to state, that an occasional case of some serious disease, in dormant localities, is very beneficial, as it arouses the inhabitants from their wonted lethargy, and tends to produce an unusual state of prolonged cleanliness. A woful exemplification of human carelessness is shown by the number of deaths which are annually caused by preventable diseases. "Ignorance is no excuse in the eyes of the law." If this phrase holds good in sanitary laws, our crimes must then be many indeed. How many fathers, mothers, sisters, brothers have stood at the bedside of some dear afflicted one, whose full blown blaze of life, had been reduced to a dim and flickering ember! How many mothers have raised their hands, with the most appealing prayers, for the life of some poor dying one! But the obnoxious surroundings were allowed to remain unaltered.

